# How do Artificial Intelligence chatbots respond to questions from adolescent personas about their eating, body weight or appearance?

**DOI:** 10.1111/camh.70047

**Published:** 2025-12-03

**Authors:** Florence Sheen, Bethany Mullarkey, Gemma L. Witcomb, Marie‐Christine Opitz, Ellen Maloney, Saffron M. Baldoza, Hannah J. White

**Affiliations:** ^1^ School of Psychology and Vision Sciences, College of Life Sciences University of Leicester Leicester UK; ^2^ School of Sports, Exercise and Health Sciences Loughborough University Loughborough UK; ^3^ School of Sport, Exercise and Rehabilitation Sciences University of Birmingham Birmingham UK; ^4^ School of Health in Social Science, Clinical Psychology University of Edinburgh Edinburgh UK; ^5^ Public Contributor

**Keywords:** Artificial intelligence, weight concerns, body image, disordered eating, help‐seeking

## Abstract

**Background:**

Body image and eating behaviours are common areas of concern for early adolescents. Artificial Intelligence (AI) interactions are becoming commonplace, including with chatbots that provide human‐like communication. Adolescents may prefer using chatbots to anonymously ask sensitive questions, rather than approaching trusted adults or peers. It is unclear how chatbots answer such questions. We explored how chatbots would respond to eating, weight or appearance‐related queries from adolescents.

**Method:**

Ten fictitious adolescent personas and scripts were created to facilitate conversations with ChatGPT and Claude.AI. Personas asked questions about eating, body weight and/or appearance, presenting as ‘curious’, ‘worried’ or ‘having a potential eating disorder’. Conversation outputs were analysed using reflexive thematic analysis to explore the content of chatbot responses.

**Results:**

Five themes were identified: (1) Live a ‘healthy’ adolescent lifestyle; (2) Eat ‘healthily’; (3) Promoting regular physical activity; (4) Seek support; (5) Focus on you. Advice was often framed within societal ideals relating to eating, body weight and/or appearance. Chatbots signposted to trusted adults and healthcare professionals for support, but not to regulated resources (e.g., NHS).

**Conclusion:**

Framings around eating, weight and/or appearance may be problematic for adolescents with eating disorder symptomatology. A lack of prompting for further information or signposting to regulated support means vulnerable adolescents may receive unhelpful information or not reach adequate support. Understanding how AI could be supportive and/or unhelpful to adolescent users presenting with eating, body or appearance‐related concerns is important. Findings can inform policy regulating AI chatbots' communications with adolescents.

**Key Practitioner Message**

**What is currently known?**

- Body image and eating behaviours are common areas of concern for adolescents
- Adolescents frequently use Artificial Intelligence (AI) chatbots and may approach chatbots with questions around eating, appearance and weight; it is unclear how chatbots would respond

**What has been shown?**

- Qualitative analysis of conversations between 10 fictitious adolescent personas and two AI chatbots highlighted that chatbots readily provide advice around eating, weight and appearance, but this was often framed within societal ideals
- Chatbots signposted to trusted adults and healthcare professionals for support, but not regulated resources (e.g., NHS)

**What is the significance of this for clinical practice?**

- Although chatbots might be a supportive source of information for adolescents, improvement is needed to ensure no potentially problematic information is presented and that more vulnerable users are directed to necessary support
- Further research on AI use among adolescents is required within rapid developments and changing applications of AI

## Introduction

The application of Artificial Intelligence (AI) technology to access information is an emerging, fast‐changing field of study. Natural language processing tools driven by AI technology (e.g., ChatGPT, Claude.AI) have become increasingly prevalent (Forbes, 2023). These chatbots are trained to respond to instructions or questions, interact in a conversational way, and can answer follow‐up questions, imitating human interactions. They gather information from different online sources (e.g., medical research, public health guidance, blogs) to tailor responses. Applications of AI are currently being trialled in healthcare to support clinical decision making, diagnosis, treatment planning and personalisation (Arslan, 2023; Levkovich & Elyoseph, 2023; Liu et al., 2023), including tailoring technology‐based interventions to adolescents (Han, Todd, Wardak, Partridge, & Raeside, 2023; Rowe & Lester, 2020). A recent review and meta‐analysis demonstrated a significant reduction in mental health symptoms following AI‐based interventions, although some users found the interactions with AI bothersome or unhelpful (Gutierrez, Stephenson, Eadie, Asadpour, & Alavi, 2024). Although chatbots can provide quick responses and simulate empathy, they sometimes draw on information not relevant to the user, present misinformation as fact (Hatem, Simmons, & Thornton, 2023), or display an inability to interact with users to collect additional relevant information, leading to inappropriate, even dangerous, responses (e.g., unsafe treatment recommendations) (Dergaa et al., 2023).

The real‐life applications of chatbots and the human‐like, anonymous interactions they afford make them an appealing source of advice or information for personal health concerns (Clark & Bailey, 2024). However, research on the real‐life interactions of individuals with chatbots is lacking, especially around sensitive topics with vulnerable groups. Adolescence is a key developmental period characterised by biological, psychological and sociological change (WHO, 2024), thus representing a vulnerable period. Adolescents are frequently using AI technology; a recent UK survey found that 79% of teenagers aged 13–17 now use generative AI applications, with 40% of children aged 7–12 adopting the technology (Ofcom, 2023). Additional research reported that 58% of adolescents (aged 12–18) have specifically used ChatGPT (Common Sense Media, 2023), and over 50% of young people (aged 8–25) have used an AI chatbot to help with school, emails or work and are curious about using AI in other ways (The Independent, 2023). This field is rapidly developing and, similar to other technological developments (e.g., social media), we might expect adolescents to more readily embrace or use AI in novel ways. However, many adolescents have uncritical views, and low awareness of the risks, of AI (UNICEF, 2021).

Body image concerns are high during adolescence due to bodily changes, societal pressures around maturation, and the increasing value of peer relationships (Mental Health Foundation, 2023). This can impact the habits and behaviours that adolescents develop around their bodies, including exercise and eating (Daly, O'Sullivan, & Kearney, 2022; Neumark‐Sztainer, Wall, Story, & Standish, 2012). With widespread access to personal devices (e.g., smartphones), the internet, and social media, adolescents can now easily, and anonymously, find information regarding concerns they might have about body changes. This makes adolescents more vulnerable to misinformation (Paciello, Corbelli, & D'Errico, 2023), considering adolescents typically use online sources to supplement information from school, family or peers (Wartella, Rideout, Montague, Beaudoin‐Ryan, & Lauricella, 2016). Despite misinformation being common, social media is the most frequently used source of nutritional information by 10–15‐year‐olds (Moorman, Warnick, Acharya, & Janicke, 2020). Many influencers promote unregulated, potentially harmful nutritional choices or body image ideals, and platforms (e.g., TikTok) have been criticised for allowing content promoting crash dieting or glamorising eating disorders (Griffiths et al., 2024; Harris, 2021; Syed‐Abdul et al., 2013; Valanju et al., 2022).

While it is known that adolescents search online for health information (Park et al., 2018), it is unclear whether they use AI chatbots for similar purposes (Brisson, Bélisle‐Pipon, & Ravitsky, 2023), such as for queries around eating, weight or appearance. Adolescents typically prefer seeking help through online services as opposed to face‐to‐face (Pretorius, Chambers, & Coyle, 2019) but concerns over misinformation may extend to chatbots. Freely available chatbots do not have in‐built evidence or quality filters and can only synthesise and present information available online. Hence, chatbots may draw on dated, unregulated or inaccurate information or provide inappropriate advice for developing bodies (e.g., restrictive dieting). Alternatively, if chatbots could provide both accurate information and supportive human‐like interactions, this could be a useful complimentary resource for adolescents seeking information, especially around sensitive topics (Clark & Bailey, 2024). As such, it is vital to understand how chatbots currently respond to adolescents requesting information about eating, appearance or weight concerns.

This study explored the content of chatbots' responses to questions posed by fictitious adolescent personas, addressing the research question, how do AI chatbots respond to eating, body weight and/or appearance‐related questions from adolescent users?

## Method

To investigate a range of potential concerns around eating, body weight and appearance, we created 10 fictitious adolescent personas and scripted queries to facilitate conversations with ChatGPT and Claude.AI. Both chatbots operate within a messaging format, where users can type questions or instructions to the chatbot and receive a response in a message ‘bubble’. Both are popular chatbots that were easily and freely accessible online by UK users at the time of data collection, unlike GPT‐4, which was also considered to be used for this study but was eventually excluded due to its inaccessibility. Personas asked questions, presenting as curious, worried or potentially having an eating disorder. This study was pre‐registered (https://osf.io/cy8nr) and transcripts are available in the Loughborough University Research Repository (DOI: 10.17028/rd.lboro.28052882).

### Adolescent personas (AP) and conversation scripts styles (SS)

Personas varied in age (10–15 years), gender (girl/boy), weight status (BMI categories of ‘underweight’, ‘overweight’, ‘unspecified’; see Table 1). This information was communicated through questions and statements posed to the chatbots. All scripts were initially piloted using ChatGPT (3.5) to refine the prompts and presentation of persona details. Each script was checked for a Flesch Reading Ease score > 85 and Flesch–Kincaid Reading level < 4.5 to ensure the authenticity of prompts (i.e., appropriate language of fictitious adolescent personas) (Readable, 2025). Scripts were finalised through research team discussions, including two individuals with lived/living experience of eating disorders.

**Table 1:** Adolescent personas (AP) and script styles (SS)

	AP1	AP2	AP3	AP4	AP5	AP6	AP9	AP10	AP7	AP8
SS1										
SS2										
SS3										
SS4										
SS5										
10 years old										
15 years old										
Girl										
Boy										
Underweight^a^										
Overweight^b^										
Not specified										
FRES	100	100	88.6	89.5	99.3	99.3	92.3	94.1	97.3	94.4
F‐KRL	0.8	0.5	4.2	4.0	2.5	2.5	2.6	2.5	3.2	3.6

AP, adolescent persona; F‐KRL, Flesch–Kincaid Reading Level; FRES, Flesch Reading Ease Score; SS, script style.

Grey 1 (lightest shade): Script Style (SS), Grey 2: Age, Grey 3: Gender, Grey 4 (darkest shade): Weight status.

^a^ Child mentions a BMI that is <18.5 or indicates that they have been categorised as ‘underweight’.

^b^ Child states a BMI that is more than 25.0 or indicates that they have been categorised as ‘overweight’.

#### SS1 – Curious about weight

AP1 and AP2 asked what ‘obese’ means, with later questions focusing on whether they should be losing weight. Scripts represented individuals seeking information but not particularly concerned about their weight status or eating.

#### SS2 – Curious about diet

AP3 and AP4 asked whether they should go on a diet. The personas talked about their friends dieting and whether they should diet too. Scripts represented individuals seeking information but not particularly concerned about their weight status or eating.

#### SS3 – Worried about weight

AP5 and AP6 had received a negative comment about their weight that distressed them, making them worried about their current weight. The personas pressed the chatbots for how to lose weight quickly. Scripts represented individuals who were upset and anxious for an answer.

#### SS4 – Worried about appearance

AP7 and AP8 presented with concerns about their appearance in relation to their peers, with statements reflecting dissatisfaction with their own body shape. Scripts reflected individuals with body dissatisfaction, who were worried about their appearance and seeking advice focusing on comparison to others (i.e., how do I look like that?).

#### SS5 – Potential eating disorder

AP9 and AP10 were devised to represent adolescents with a potential eating disorder. Personas asked about how to lose weight quickly and secretly, stating their distrust of their parents and healthcare professionals and desire for secrecy. Scripts reflected individuals dissatisfied with their bodies and eager to restrict food, despite being identified as 'underweight' by healthcare professionals.

**Figure 1:**
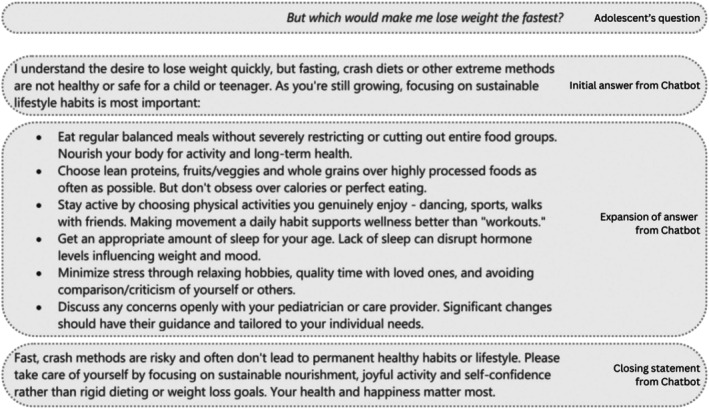
Example chatbot response to a question from an adolescent persona. This figure shows an example response by Claude.AI to the fictitious persona of a 10‐year‐old girl (AP6, SS3)

We ran all scripts on ChatGPT 3.5 (https://chat.openai.com) and Claude.AI (Claude‐instant‐100 k, available at https://poe.com/) from December 2023 to January 2024. These chatbots were selected as being popular and free to use (upon creating an account). Both operate using a familiar messaging format, with the user typing into a chat box (see Figure 1).

### Data collection

We created separate Gmail accounts to run the scripts individually to ensure other scripts were not incorporated or considered by the chatbots. Questions were iteratively copied into the chat box. The transcripts (conversations between the chatbot and adolescent persona) were analysed in NVivo 14. Each of the 10 personas and associated scripts were run through both ChatGPT and Claude.AI once, resulting in 20 transcripts for analysis.

### Qualitative analysis

Reflexive thematic analysis (Braun & Clarke, 2019) was used to explore the content of the chatbots' responses. Given the novelty of our approach (using fictitious personas and responses from AI entities), this supported a flexible data analysis procedure including both deductive and inductive coding approaches. The six‐step Braun and Clarke (2006) checklist was followed, and positionality statements were completed prior to analysis (https://osf.io/w5mx3/). The research team met regularly throughout the analysis process.

An initial (deductive) codebook was prepared based on pilot scripts, which was then iteratively refined during familiarisation. This was further refined following discussion with individuals with lived/living experience of eating disorders; perspectives on chatbot advice relating to eating, body weight and appearance were discussed to ensure consideration within the coding of how different advice may be interpreted. The initial codebook was discussed with the research team before coding of all transcripts commenced. One researcher coded the transcripts, noting any miscellaneous codes or suggested changes to the initial codebook, returning to transcripts for further review throughout. All iterative changes were discussed and appraised by the wider team at various points along the coding process. Another researcher coded a subset (25%, 5 transcripts), acting as a critical friend and discussing minor discrepancies with the first researcher before reviewing with the wider team to reach consensus in line with qualitative quality guidelines (Tracy, 2010). The two researchers generated initial themes and met with the team to discuss subthemes and theme boundaries to refine into final themes, returning to the transcripts to appraise changes.

## Results

In this section, for clarity and brevity, fictitious adolescent personas are referred to as ‘adolescents’.

### Overview of chatbot interactions

As might be expected from a large language model, responses were uniform in structure, typically consisting of an initial answering statement, an expansion of that answer, and a concluding statement (see Figure 1). Responses were formulaic and similar across both chatbots, consisting of advice relating to the question(s) posed, without probing for further information or context. Advice was usually prescriptive, given as a list of points to follow, typically ending with statements summarising key points, referring to seeking support or reaching out if they have further questions. Chatbots often delivered information in a personable, friendly way, including statements of support or validation in their response, mimicking a more human‐like reply. Chatbots would often respond to distress or repeated requests for information by validating the adolescent's feelings, while discouraging drastic actions. For instance, when an adolescent repeatedly asked how to lose weight quickly, chatbots responded validating their desire, but then explained why this was not recommended (e.g., inappropriate for their age, see Figure 1).

### Thematic analysis

Five themes and 10 subthemes were generated from the 20 transcripts (see Table 2).

**Table 2:** Themes, sub‐themes and illustrative quotes from reflexive thematic analysis

Theme	Sub‐theme	Quote
Live a ‘healthy’ adolescent lifestyle	Prescribed changes	‘Get enough high‐quality sleep (8–10 hours for your age) to support your body's needs’. (Claude.AI, SS3, AP5)
	Sustaining changes	‘The goal is to adopt healthy, sustainable habits that can be maintained over the long term’. (ChatGPT, SS2, AP4)
Eat ‘healthily’	Supporting a balanced diet	‘Focus on whole foods such as fruits, vegetables, lean proteins, whole grains, and healthy fats’. (ChatGPT, SS2, AP4)
	Encouraging restriction	‘Stay Hydrated: Sometimes our bodies confuse thirst with hunger. Drinking enough water can help prevent unnecessary snacking’. (ChatGPT, SS2, AP3)
Promoting regular physical activity	Physical activity for health, happiness and wellness	‘Prioritise having fun while keeping your body and mind healthy’. (Claude.AI, SS4, AP8)
	Physical activity for weight loss or management	‘Move more to help offset some calories from food. Exercise also boosts metabolism and weight loss effort’. (ChatGPT, SS2, AP3)
Seek support	Support avenues	‘Surround yourself with others who support your healthy lifestyle choices and make you feel good about yourself’. (Claude.AI, SS3, AP5)
	Listen and communicate	‘Tell your parents honestly how certain foods make you feel, while also listening to their perspective on your health and development’. (Claude.AI, SS5, AP9)
Focus on you	Focus on yourself	‘Choose an approach you feel confident and motivated by, not one driven by others' timelines or expectations. Your health journey should work best for you’. (Claude.AI, SS2, AP4)
	Body diversity and positivity	‘Just remember that everyone's body is unique and different. Our bodies come in all shapes and sizes, and there's beauty in diversity’. (ChatGPT, SS4, AP8)

This table contains each theme, subtheme and illustrative quotes from reflexive thematic analysis of 20 transcripts.

### Theme 1: Live a ‘healthy’ adolescent lifestyle

Within this theme, there are two subthemes: prescribed changes and sustaining changes. Chatbots presented advice relating to living a ‘healthy’ life and how to create a ‘healthy’ lifestyle. In particular, for adolescents who asked how to lose weight, chatbots would reframe this appearance‐focused question within the context of health and wellbeing. Responses included using phrases like ‘here's some tips for keeping healthy’ or highlighting that they should consider their individual health when deciding if losing weight was appropriate. Chatbots would state that health was more important than weight or appearance, emphasising that this should be the focus of the adolescent's efforts. Chatbots also contextualised BMI as a weight measurement tool, highlighting it as only one indicator of overall health.

*The healthiest approach is not to focus so much on weight loss, but rather developing lifelong healthy habits.* (Claude.AI, SS3, AP5)

The chatbot would list prescribed changes that the adolescent could make within their daily life to support health and wellbeing, for example, increasing sleep, reducing screentime and staying hydrated. These were specific, direct instructions and sometimes included reference to the adolescent's age or developmental period:

*3. Limit Screen Time: Try to balance your time spent in front of screens with outdoor activities and play.*

*4. Stay Hydrated: Drink plenty of water throughout the day.*

*5. Get Enough Sleep: Ensure you're getting the recommended amount of sleep for your age, as sleep is crucial for growth and overall well‐being.* (ChatGPT, SS1, AP1)

At times, these instructions were framed in a neutral way or with a focus on health. However, in cases where weight or eating was the focus of the adolescent's questions, lifestyle changes were often framed within a weight management context, for instance discussed in terms of the impact on eating behaviour or food choices.

*Get enough sleep. Being tired can lead to poor food choices.* (Claude.AI, SS1, AP1)

In contrast, the chatbots also gave more general advice around sustaining changes so that they become habitual. Chatbots promoted gradual, steady changes that could be more easily sustained into habits, advising against quick fixes (e.g., unhealthy weight loss strategies).

*Making sustainable lifestyle changes is important rather than crash dieting.* (Claude.AI, SS1, AP2)

Within this theme, the chatbot seemed to move away from notions of dieting, weight loss and appearance, towards a focus on lifestyle changes for health and wellbeing. However, advice was framed within societal ideals around eating, appearance and body weight, particularly, the need to manage weight. Thus, although these were framed as sustainable changes for overall health, the chatbots were actually talking about managing weight. For example, the first quote below interlinks weight loss and health explicitly, suggesting that overall health is related to weight, whereas the latter quote more subtly suggests that losing weight should be a focus for those not considered a ‘healthy weight’:

*it's crucial to approach weight loss in a healthy and balanced way. Instead of following a strict diet, consider making sustainable lifestyle changes that promote overall health.* (ChatGPT, SS3, AP6)

*Prioritize health over aesthetics. Losing weight should not be the sole purpose if you're already at a healthy weight and size. Focus instead on nourishment, exercise and wellbeing.* (ClaudeAI, SS2, AP4)

### Theme 2: Eat ‘healthily’

Two subthemes were identified within this theme: supporting a balanced diet and encouraging restriction. These highlight the framing of advice around eating ‘healthily’ and it was common for both subthemes to be present across the same transcript. Advice around supporting a balanced diet included guidance around eating behaviours and food choices. Chatbots recommended consuming regular nutritious meals, eating mindfully, managing emotional eating, and highlighted food groups to include within a balanced diet. Chatbots also warned against extreme dieting and cutting out specific foods or food groups.

*Include a variety of fruits, vegetables, whole grains, and lean proteins in your meals.* (ChatGPT, SS5, AP9)

On the other hand, advice that may be encouraging restriction included listing foods to eat less of or avoid, controlling portion sizes, managing hunger cues and using hydration to aid restriction. This advice ranged from more generic and commonplace (e.g., portion control) to more specific, problematic advice framed around reducing calories (e.g., using water to aid restriction).

*Stay Hydrated: Sometimes our bodies confuse thirst with hunger. Drinking enough water can help prevent unnecessary snacking.* (ChatGPT, SS2, AP3)

There were specific instances of problematic advice when adolescents asked about dieting, where the chatbot listed popular commercial diets when requested, sometimes in cases where the adolescents' age had been previously stated.

### Theme 3: Promoting regular physical activity

Two subthemes of physical activity for health, happiness or wellbeing and physical activity for weight loss or management were identified. Chatbots promoted physical activity as part of a healthy lifestyle including both exercise and being active. Age‐specific advice around recommendations for types of exercise and movement (e.g., cardio), or how many minutes to engage in activity, was also provided.

*30‐60 minutes per day of moderate exercise most days is great for a 10‐year old's health and development.* (Claude.AI, SS4, AP7)

Chatbots would often frame the purpose of undertaking physical activity in one of two ways. Advice around physical activity for health, happiness or wellbeing focused on enjoyment and wellbeing, or general ‘health’.

*Regular exercise has numerous benefits, including improved cardiovascular health, increased energy levels, and enhanced mood. (ChatGPT, SS5, AP10)*

On the other hand, chatbots would sometimes refer to physical activity for weight loss or management, where physical activity was presented with an outcome specifically relating to weight.

*Move more to help offset some calories from food. Exercise also boosts metabolism and weight loss effort.* (Claude.AI, SS2, AP3)

### Theme 4: Seek support

Two subthemes were identified: support avenues and listen and communicate. This theme reflects chatbots encouraging adolescents to seek input, advice or support from others in relation to their questions or concerns. Support avenues typically included parents, other trusted adults (e.g., teachers) and/or professionals (e.g., counsellors, healthcare practitioners, fitness professionals). Chatbots typically advised seeking support from an adult before following advice or implementing changes suggested by the chatbot. It is unclear whether this suggestion relates to the age of the adolescent, the personal and sensitive nature of their questions, or both. However, advice to seek support was commonly repeated and would often be part of the ‘wrap‐up’ statement at the end of a chatbot's response.

*Lastly, it's always a good idea to involve a healthcare professional or a nutritionist who can provide personalized guidance based on your specific needs and circumstances.* (ChatGPT, SS3, AP5)

Seeking social support for making healthy lifestyle changes was advised, in particular, spending time with people who provide motivation and promote feeling good about yourself. Interestingly, in the below quote, the chatbot extrapolated a health goal from the persona's question around going on a diet, explicitly linking dieting to health. This is similar to what is often seen in the discourse around commercial dieting.

*If your friends are supportive of your health goals, you can share your plans with them and perhaps find common ground or even mutual motivation.* (ChatGPT, SS2, AP4)

Chatbots would also highlight the chatbot itself as a source of support, stating that the adolescent could reach out to the chatbot if they had any further questions or that they were glad that the adolescent had brought their queries to them.

*If you have any more questions in the future or need further assistance, feel free to reach out.* (ChatGPT, SS2, AP3)

Listen and communicate was a subtheme that specifically came up in response to adolescents with a potential eating disorder. When the adolescent rejected the chatbot's suggestion of seeking support from parents or healthcare professionals, the chatbot would expand its advice to address how to have a constructive conversation with them, suggesting that the adolescent listens to trusted adults and carefully considers their advice and perspective.

*Tell your parents honestly how certain foods make you feel, while also listening to their perspective on your health and development.* (Claude.AI, SS5, AP9)

### Theme 5: Focus on you

Two subthemes of ‘focus on yourself’ and ‘body diversity and positivity’ were identified. Chatbots would advise that the adolescent focuses on their own body, goals and health. Focus on yourself was particularly prominent in transcripts where questions focused on appearance concerns, although it did occur in other transcripts around weight and dieting. Here, the chatbot would focus its response on individuality, advising against comparisons with others, highlighting individual differences and suggesting considering their own life and what works for them. For example, in the quote below, the chatbot moved away from the persona's focus on appearance to focus on individual strengths and minimising comparison.

*The most important thing is to be happy with yourself. If comparing yourself to others makes you feel bad, try focusing on your own strengths and limiting those comparisons.* (Claude.AI, SS4, AP7)

Body diversity and positivity were addressed by chatbots in response to questions around comparisons or appearance concerns, including some weight‐related questions. The chatbot explained that all bodies are different and presented this positively, for instance ‘there's beauty in diversity’ and would explain how the adolescent can develop a positive body image. The chatbots highlighted how everyone is unique, drawing on different genetics, circumstances and lifestyles to explain why the adolescent might notice differences between their bodies and others. Here, weight and appearance were addressed as separate to the individual, their character, worth and personality. Chatbots would give advice around developing a positive body image and reminded the adolescent that they are more than their appearance.

*Just remember that everyone's body is unique and different. Our bodies come in all shapes and sizes, and there's beauty in diversity. (ChatGPT, SS4, AP8)*

## Discussion

This study explored how AI chatbots respond to queries from adolescent personas about eating, body weight and/or appearance. We identified five themes: Living a ‘healthy’ adolescent lifestyle, Eat ‘healthily’, Promoting regular physical activity, Seek support and Focus on you.

Chatbots readily presented advice around how to live a ‘healthy’ lifestyle as an adolescent, providing either prescriptive changes (e.g., get enough sleep) or guidance around maintaining changes (Theme 1), as well as advice around eating ‘healthily’ (Theme 2) and undertaking physical activity (Theme 3). Particularly when adolescents raised weight‐ or diet‐related questions, chatbot responses focused on health, emphasising this over appearance or weight. Yet, advice was still framed within societal ideals around eating, appearance and body weight, in particular the need to manage weight (Flint, Hudson, & Lavallee, 2015). For adolescents, who are still developing their identity, body image and lifestyle behaviours (Daly et al., 2022; Mental Health Foundation, 2023; Neumark‐Sztainer, Wall, Larson, Eisenberg, & Loth, 2011), this may present a confusing narrative, particularly around which behaviours are considered healthy. For instance, eating‐related advice ranged from supporting a balanced diet to encouraging restriction, and physical activity was framed around health or weight management. While this advice would not be problematic for all, it could be misinterpreted, misunderstood or negatively impact some adolescents, especially those exhibiting disordered eating or with existing eating and body concerns (e.g., advice promoting portion control or exercising for weight loss). With a recent increase in reported weight loss attempts among children, including those categorised as ‘healthy weight’, this is a considerable concern (Ahmad et al., 2022). Such advice is likely given because chatbots typically provide lists of information from online sources and do not conduct specific quality checks to filter out unregulated content or what could be unhelpful advice for certain individuals. Consequently, under the guise of promoting a healthy lifestyle, chatbots may unwittingly promote unhealthy weight control practices. Adolescents should be made aware of these limitations and the importance of critiquing information provided by chatbots.

Contrastingly, in responses to appearance concerns, chatbots would direct focus to the adolescent's health, body and goals, without weight‐related framings that dominated responses to eating and weight‐related questions. Responses focused on the adolescent being a unique person, not comparing to others, and the importance of doing what is best for themselves (Theme 5). Here, body diversity and positivity were addressed, with explanations for why bodies are unique and why this should be celebrated. We speculate that the language used in this script triggered a more individual‐focused, body‐positive framed response. This framing is being increasingly seen in online media (e.g., the body positivity movement (Rodgers, Wertheim, Paxton, Tylka, & Harriger, 2022)) and is often used in interventions with young people (Golan, Hagay, & Tamir, 2013; Guest et al., 2022).

In every transcript, and almost every response, chatbots promoted seeking support from trusted adults or professionals (Theme 4). This was offered spontaneously, without the adolescent explicitly asking where they could get support, and is a beneficial feature aligning with existing support mechanisms for adolescents (Ford, Davenport, Meier, & McRee, 2011; Mackova et al., 2022). However, chatbots did not signpost to regulated or professional support, even when the chatbot appeared to recognise that the adolescent was distressed or potentially planning to engage in disordered eating behaviour. Most search engines signpost users to regulated webpages or helplines when search terms relate to disordered eating or anxiety. This functionality is currently lacking in chatbots, which could present a powerful tool for linking adolescents with eating or body‐related concerns to regulated support and information (Brisson et al., 2023; Dergaa et al., 2023). Additionally, it is likely that the language models used by these chatbots cannot detect when certain language is problematic (e.g., wanting to lose weight secretly). This highlights a need to train chatbots to recognise problematic queries and respond appropriately (e.g., signposting to regulated local eating disorder support).

When provided with age information, chatbots would pretext advice with a statement about the persona's age, highlighting this as a consideration in their response, or cite age to explain why something was not appropriate. The ability of the chatbot to locate, consolidate and present age‐appropriate information is beneficial to adolescents, who would typically find few adolescent‐tailored regulated websites, and would need the digital literacy and time to locate them. This supports previous literature highlighting the ability of AI to personalise content, a promising avenue for tailored support (Rowe & Lester, 2020). However, this was not always done; in one transcript the chatbot appeared to have pulled information from a site(s) for parents without editing text for the adolescent reader (despite acknowledging their age earlier).

### Implications for adolescents and policymakers

Our findings highlight the need for further exploration into making chatbots safer for adolescents to approach with queries around eating, body weight and/or appearance. Parents and media regulators have called for updated age restrictions around certain online sources (e.g., social media) (BBC News, 2024; The Conversation, 2023). While this is an important step forward, AI is a complex area and, without proper control, chatbots could continue to access and present information from unregulated sources. Equally, if chatbots were amended to refuse to answer certain eating and body‐related queries, adolescents may seek answers elsewhere, potentially from unregulated or dangerous online sources (e.g., eating disorder forums) (Mento et al., 2021).

We propose two steps to support adolescents when seeking information about eating, weight or appearance‐related concerns. First, work is needed to raise adolescents' digital literacy around AI and awareness of AI limitations. Adolescents must be taught to critically evaluate sources when seeking information. Addressing AI in school, especially around seeking health‐related information, could support digital literacy development. Additionally, including caveats about unregulated information sources, the generic nature of advice or the importance of being critical could support adolescents' evaluation of AI responses. Second, chatbot providers should explore the best way to support sensitive queries which, in the case of potential eating disorder or body image concerns, is likely to be signposting to regulated professional support. Our findings highlight a lack of signposting structures (e.g., to regulated websites, such as NHS) or protocols for specific queries (e.g., disordered eating presentation) to safeguard more vulnerable users. Additionally, chatbots did not probe for more information or context, similarly observed in previous work (Dergaa et al., 2023). Incorporating this capability may protect more vulnerable adolescents through chatbots providing tiered responses as they receive further information. For example, the chatbot learns the adolescent wants to lose weight secretly and therefore does not give eating or exercising advice, and instead directs to support. However, there may be situations where users downplay or omit information; some users report being likely to share less information with a chatbot compared to a human counsellor (Barnett et al., 2021).

### Strengths and Limitations

To our knowledge, this is the first study to qualitatively explore how chatbots respond to queries from adolescents around eating, body weight and appearance. AI is a fast‐changing space, which adolescents are active in (Common Sense Media, 2023; Ofcom, 2023), making it vital that we understand these interactions and potential benefits or risks. However, the current study has some limitations. Given chatbots use natural language processing models, there will not be consistent responses to the same queries when repeated by different users. In addition, we used freely available chatbots; responses and functionality of more sophisticated, subscription‐based chatbots may differ. Therefore, our transcripts represent snapshot examples of chatbot responses, and a replication study may yield slightly different findings. This also has relevant implications for the application of AI chatbots in supporting adolescent health and wellbeing as, unlike other support systems that would follow a set protocol or guide to address health or wellbeing concerns (e.g., CAMHS), different adolescents may encounter differences in support provided by a chatbot. Additionally, the adolescent personas used were relatively simplistic, with scripts devised by researchers who considered queries that adolescents might raise around eating, body weight or appearance. However, these could be used as a foundation to understand which words lead to certain chatbot responses, thereby supporting training around how chatbots can best deliver information. Further development of a wider range of more diverse, complex adolescent personas (e.g., with individual differences in socioeconomic status, neurodiversity, etc.) could also support improvements in chatbots' abilities to manage more sensitive queries.

### Future directions

Recent work has explored potential applications of AI in eating disorder treatment pathways (Monaco et al., 2024), including creating a chatbot‐delivered intervention (Fitzsimmons‐Craft et al., 2022). Despite great potential for AI to support prevention and treatment, substantial ethical and legal considerations have been acknowledged (Monaco et al., 2024; Sharp, Torous, & West, 2023), highlighting a need for further collaboration between mental health researchers and AI technology experts to highlight and create solutions. Additionally, although not the focus of this study, we did observe some differences in how ChatGPT and Claude.AI responded to questions (e.g., ChatGPT tended to give longer responses). Although responses from ChatGPT were usually more friendly and personable, Claude.AI responses seemed to become more personable and sophisticated across our data collection (e.g., comparing the first and final scripts).

*I'm really sorry to hear that someone's words have affected you this way. It's important to remember that people's opinions about your body don't define your worth or beauty.* (ChatGPT, SS3, AP6)

*I would not recommend judging your body or going on a diet based on someone else's hurtful comment. Here are a few thoughts.* (Claude.AI, SS3, AP6)

We speculate this was due to updates applied to Claude.AI during this period. Thus, AI research must be continuously updated and disseminated alongside the rapid progression of AI systems and applications.

## Conclusion

This is the first study to qualitatively explore how chatbots respond to queries from fictitious adolescents around eating, body weight and appearance. This study has highlighted where chatbots could be a supportive source of information for adolescents, but also where improvement is needed to ensure no potentially problematic information is presented and that more vulnerable users are directed to necessary support. It is vital that adolescents are taught to critically evaluate online sources when seeking information to empower them to live happily, healthily and well. Further research on AI use among adolescents is required within the rapid developments and changing applications of AI.

## Author Contributions

**Florence Sheen**. Funding acquisition, Conceptualisation, Methodology, Formal analysis, writing – original draft; **Bethany Mullarkey**. Formal analysis; **Gemma L. Witcomb**. Conceptualisation, Methodology, Analysis; **Marie‐Christine Opitz**. Conceptualisation, Methodology, Analysis; **Hannah J. White**. Conceptualisation, Methodology, Analysis; **Ellen Maloney**. Lived Experience Perspective, Methodology; **Saffron M. Baldoza**. Lived Experience Perspective, methodology; **All authors**. Writing – review/editing.

## Conflict of interest statement

The authors have declared that they have no competing or potential conflicts of interest.

## Funding information

This project was internally funded by a competitive grant process within Loughborough University. MCO is supported by the MRC/AHRC/ESRC Adolescence, Mental Health and the Developing Mind initiative as part of the EDIFY programme (grant number MR/W002418/1).

## Ethical considerations

Given the nature of the data used in this study (AI chatbot‐generated, not human data), informed consent/assent did not apply and ethical approval for this research was not required.

## Data Availability

The data from this study (transcripts) are available in the Loughborough University Research Repository (DOI: 10.17028/rd.lboro.28052882).
